# Pentacyclic Nitrofurans with *In Vivo* Efficacy and Activity against Nonreplicating *Mycobacterium tuberculosis*

**DOI:** 10.1371/journal.pone.0087909

**Published:** 2014-02-05

**Authors:** Rakesh, David F. Bruhn, Michael S. Scherman, Lisa K. Woolhiser, Dora B. Madhura, Marcus M. Maddox, Aman P. Singh, Robin B. Lee, Julian G. Hurdle, Michael R. McNeil, Anne J. Lenaerts, Bernd Meibohm, Richard E. Lee

**Affiliations:** 1 Department of Chemical Biology and Therapeutics, St. Jude Children’s Research Hospital, Memphis, Tennessee, United States of America; 2 Mycobacterial Research Laboratories, Department of Microbiology, Colorado State University, Fort Collins, Colorado, United States of America; 3 Department of Pharmaceutical Sciences, College of Pharmacy, University of Tennessee Health Science Center, Memphis, Tennessee, United States of America; 4 Biomedical Sciences Program, Graduate Health Sciences, University of Tennessee Health Science Center, Memphis, Tennessee, United States of America; The Scripps Research Institute and Sorrento Therapeutics, Inc., United States of America

## Abstract

The reductively activated nitroaromatic class of antimicrobials, which include nitroimidazole and the more metabolically labile nitrofuran antitubercular agents, have demonstrated some potential for development as therapeutics against dormant TB bacilli. In previous studies, the pharmacokinetic properties of nitrofuranyl isoxazolines were improved by incorporation of the outer ring elements of the antitubercular nitroimidazole OPC-67683. This successfully increased stability of the resulting pentacyclic nitrofuran lead compound Lee1106 (referred to herein as **9a**). In the current study, we report the synthesis and antimicrobial properties of **9a** and panel of **9a** analogs, which were developed to increase oral bioavailability. These hybrid nitrofurans remained potent inhibitors of *Mycobacterium tuberculosis* with favorable selectivity indices (>150) and a narrow spectrum of activity. *In vivo*, the pentacyclic nitrofuran compounds showed long half-lives and high volumes of distribution. Based on pharmacokinetic testing and lack of toxicity *in vivo,* **9a** remained the series lead. **9a** exerted a lengthy post antibiotic effect and was highly active against nonreplicating *M. tuberculosis* grown under hypoxia. **9a** showed a low potential for cross resistance to current antitubercular agents, and a mechanism of activation distinct from pre-clinical tuberculosis candidates PA-824 and OPC-67683. Together these studies show that **9a** is a nanomolar inhibitor of actively growing as well as nonreplicating *M. tuberculosis*.

## Introduction

*Mycobacterium tuberculosis* remains an important global pathogen that is believed to have infected a third of the world’s population and kills over a million persons annually [1]. The US Centers for Disease Control and Prevention recommended regime for drug sensitive tuberculosis (TB) infections consists of rifampin, isoniazid, pyrazinamide and ethambutol for an initial two months followed by continued four month treatment with isoniazid and rifampin. Poor patient compliance to this lengthy drug regimen can lead to patient relapse and promotes the emergence of drug-resistant strains, which have become a major treatment challenge and global health threat. Tuberculosis strains resistant to isoniazid and rifampin, two of the most powerful antituberculosis agents, are classified as multi drug-resistant (MDR TB). Extensively drug-resistant (XDR) strains, which are additionally resistant to a fluoroquinolone, and an injectable second-line drug, have been reported in over 80 countries [2]. MDR/XDR TB dramatically increases the medical burden, requiring extended treatment lengths with drugs that are less effective and show significant toxicity [3], [4]. It has been widely postulated that the key to decreasing treatment times and the spread of drug-resistant tuberculosis is the development of new therapeutics that target dormant bacilli, which are metabolically inactive cells that display phenotypic (non-genetic) resistance to most drugs [5]. Dormant bacteria are believed to reside within the nutrient and oxygen limited environment of necrotic lesions. Therefore, it is imperative that novel antitubercular agents target these persisting bacterial subpopulations [6], [7].

**Figure 1:**
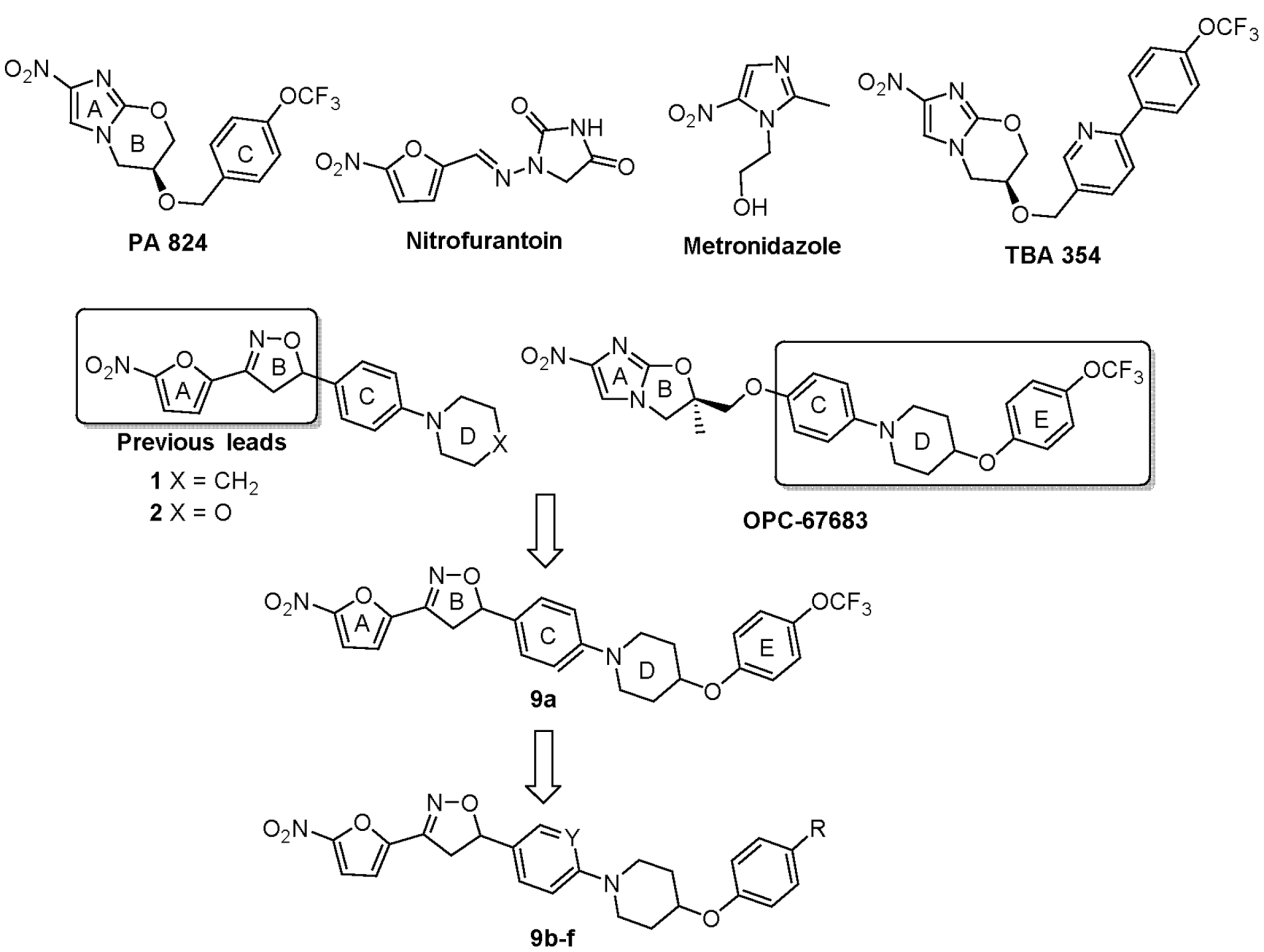
Known nitroaromatic antimicrobial drugs and nitrofurans explored in this study.

Nitroaromatic antibiotics, such as metronidazole and nitrofurantonin, are widely used to treat anaerobic bacterial infections (Figure 1). Based on this knowledge, researchers at the PathoGenesis Corporation developed a nitroimidazole pyran, PA-824 (Figure 1), with specific anti-tuberculosis activity against both actively growing and hypoxic tuberculosis bacilli [8]. PA-824 is structurally related to metronidazole and was selected for its specificity in terms of antitubercular activity, favorable metabolic stability, low toxicity profile, and lack of cross-resistance with other TB drugs. PA-824 has advanced into Phase II combination clinical testing by the Global Alliance for TB Drug Development [8], [9], [10], [11]. Researchers at Otsuka Pharmaceutical Company subsequently developed the nitroimidazole oxazole OPC-67683 (Figure 1), which is now being progressed to Phase III clinical trials against tuberculosis [12], [13], [14]. These studies highlight the potential for the successful development of antitubercular nitroaromatics.

In addition to the nitroimidazoles, other nitroaromatics belonging to nitrothiazole and nitrofuran classes have notable antitubercular activity [8], [12]. This includes our recently reported series of tetracyclic nitrofuranyl isoxazolines, which had improved solubility and bioavailability but decreased potency and metabolic stability compared to precursor nitrofurans [15], [16]. Key differences in these compounds arise from differences in their redox potential, which drive how these compounds are activated and the types of reactive products that are produced resulting in different modes of cell killing [17]. Therefore all three classes of nitroaromatics have the potential to be simultaneously developed as TB treatments, as shown in this study. Previously, incorporation of the outer ring elements of OPC-67683 into tetracyclic nitrofuranyl isoxazolines produced a novel pentacyclic hybrid nitrofuran with improved metabolic stability but limited oral bioavailability, Lee1106 (herein referred to as compound **9a**) [**18**]. Herein, we report the synthesis and evaluation of **9a** and a series of **9a**-analogs with improved *in vivo* exposure profiles (area under the curve, AUC). Of the pentacyclic hybrid nitrofurans reported, **9a** was the most metabolically stable and did not show any adverse effects *in vivo*. Therefore, **9a** remained the most promising lead candidate and was subjected to a detailed antimicrobial characterization. **9a** was highly active in two separate *in vitro* models of nonreplicating tuberculosis, was active in a murine model of acute tuberculosis infection, and was activated by a mechanism distinct from that required for PA-824 and OPC-67683.

## Materials and Methods

### Reagents and Instrumentation

All anhydrous solvents and starting materials were purchased from Aldrich Chemical Co. (Milwaukee, WI). All reagent grade solvents used for chromatography were purchased from Fisher Scientific (Suwanee, GA) and flash column chromatography silica cartridges were obtained from Biotage Inc. (Lake Forest, VA). The reactions were monitored by thin-layer chromatography (TLC) on pre-coated Merch 60 F_254_ silica gel plates and visualized using UV light (254 nm). A Biotage FLASH column chromatography system was used to purify mixtures. All NMR spectra were recorded on a Bruker-400 spectrometer. Chemical shifts (δ) are reported in parts per million relative to the residual solvent peak or internal standard (tetramethylsilane), and coupling constants (*J*) are reported in hertz (Hz). High resolution mass spectra were recorded on a Waters Xevo G2 QTOF LCMS using ESI. Purity of the products was confirmed before testing by analytical RP-HPLC on a Shimadzu HPLC system, and all final compounds had a purity of 95% or greater as determined by RP-HPLC. Gradient Conditions: solvent A (0.1% formic acid in water) and solvent B (0.1% formic acid in acetonitrile): 0–1.00 min 95% A, 1.00–6.00 min 0–95% B (linear gradient), 6.00–9.50 min 100% B, 9.50–9.75 min 0–95% A, 9.75–10.0 min 95% A, detection by UV at 254 nm and by ELSD. The reference compound PA-824 was synthesized in six step sequence following the procedure reported by Baker et al., starting from 2,4-dinitro, 1*H*-imidazole and S-glycidol [19]. OPC-67683 was synthesized from 2-chloro-4-nitro-1*H*-imidazole as described by Tsubouchi et al. [20].

### Synthesis of *tert*-Butyl 4-((methylsulfonyl)oxy)piperidine-1-carboxylate 4

Et_3_N (10.38 mL, 74.62 mmol) was added to a stirred and cooled (0°C) solution of *tert*-butyl 4-hydroxypiperidine-1-carboxylate **3** (5 g, 24.87 mmol) in CH_2_Cl_2_ (50 mL), followed by methanesulfonyl chloride (2.88 mL, 37.31 mmol) drop wise and stirred at the same temperature for 1 h. The reaction was then quenched with ice-cold water (20 mL), washed with water (20 mL), dried over anhydrous Na_2_SO_4_, and concentrated under reduced pressure to afford **4** (6.52 g) in 94% yield. ^1^H NMR (500 MHz, CDCl_3_): δ 4.72–4.82 (m, 1H), 3.29–3.39 (m, 4H), 3.16 (s, 3H), 1.52–1.77 (m, 4H), 1.38 (s, 9H); LCMS: 280 (M^+^+1).

### General Procedure I, for Preparation of Compounds 5a–b

A mixture of appropriate phenol (1 mmol), **4** (1.5 mmol), K_2_CO_3_ (3 mmol) and tetra *n*-butyl ammonium chloride (0.2 mmol) in H_2_O was heated to reflux overnight. The reaction mass was then cooled to room temperature and the product was extracted with CH_2_Cl_2_ twice. The combined organic layers were washed with water, 10% NaOH, water, dried over anhydrous Na_2_SO_4_ and concentrated under reduced pressure to afford **5a–b** in 86–90% yields.

*tert*-Butyl 4-(4-(trifluoromethoxy)phenoxy)piperidine-1-carboxylate **5a:** 4-Trifluoromethoxy phenol (3 g, 16.85 mmol), **4** (7 g, 25.28 mmol), K_2_CO_3_ (6.97 g, 50.56 mmol) and tetra *n*-butyl ammonium chloride (0.933 g, 3.37 mmol) were stirred in H_2_O and the reaction was carried out as described in general procedure I to afford **5a** (5.5 g) in 90% yield. ^1^H NMR (500 MHz, CDCl_3_): δ 6.78–6.82 (m, 4H), 3.72–3.75 (m, 1H), 3.29–3.42 (m, 4H), 1.78–2.02 (m, 4H), 1.37 (s, 9H); LCMS: 362 (M^+^+1).

*tert*-Butyl 4-(4-fluorophenoxy)piperidine-1-carboxylate **5b:** 4-Fluorophenol (1.3 g, 11.60 mmol), **4** (4.86 g, 17.39 mmol), K_2_CO_3_ (4.81 g, 34.8 mmol) and tetra *n*-butyl ammonium chloride (0.645 g, 2.31 mmol) were stirred in H_2_O and the reaction was carried out as described in general procedure I to afford **5b** (2.9 g) in 86% yield. ^1^H NMR (500 MHz, CDCl_3_): δ 7.13 (d, *J* = 2.8 Hz, 2H), 7.08 (d, J = 3.4 Hz, 2H), 3.66–3.70 (m, 1H), 3.29–3.39 (m, 4H), 1.75–2.00 (m, 4H), 1.38 (s, 9H); LCMS: 296 (M^+^+1).

### General Procedure II, for Preparation of Compounds 6a–b

Compounds **5a–b** were dissolved in 1∶1 CH_2_Cl_2_ and trifluoroacetic acid (TFA) and stirred at room temperature for 1 h and concentrated under reduced pressure, washed with water, 10% NaOH and water, dried over anhydrous Na_2_SO_4_ and concentrated to afford amines **6a–b** in 92–95% yields.

4-(4-(Trifluoromethoxy)phenoxy)piperidine **6a:** Compound **5a** (5 g, 13.85 mmol) was dissolved in CH_2_Cl_2_ and TFA (1∶1, 20 mL) and the reaction was carried out as described in general procedure II to afford **6a** (3.43 g) in 95% yield. ^1^H NMR (500 MHz, CDCl_3_): δ 6.84–7.02 (m, 4H), 3.68–3.71 (m, 1H), 2.68–2.78 (m, 4H), 1.72–1.98 (m, 4H); LCMS: 262 (M^+^+1).

4-(4-Fluorophenoxy)piperidine **6b:** Compound **5b** (2.6 g, 8.80 mmol) was dissolved in CH_2_Cl_2_ and TFA (1∶1, 10 mL) and the reaction was carried out as described in general procedure II to afford **6b** (1.58 g) in 92% yield. ^1^H NMR (500 MHz, CDCl_3_): δ 7.09–7.15 (m, 4H), 3.70–3.72 (m, 1H), 2.69–2.77 (m, 4H), 1.73–2.00 (m, 4H); LCMS: 196 (M^+^+1).

### General Procedure III, for Preparation of 7a–f

A mixture of aryl bromide (1.0 mmol), **6a** (for compounds **7a–c**) or **6b** (for compounds **7d–f**) (1.2 mmol), diacetoxypalladium (0.2 mmol), sodium butan-1-olate (2.4 mmol) and 2-(di-*tert*-butylphosphino)biphenyl (0.4 mmol) were stirred in anhydrous toluene and heated to 80°C for 3 h, then the reaction mass was concentrated under reduced pressure and purified by flash chromatography to afford **7a–f** in 50–70% yields.

4-(4-(Trifluoromethoxy)phenoxy)-1-(4-vinylphenyl)piperidine **7a:** 1-Bromo-4-vinylbenzene (1.0 g, 5.46 mmol), **6a** (1.71 g, 6.55 mmol), diacetoxypalladium (0.24 g, 1.09 mmol), sodium butan-1-olate (1.26 g, 13.11 mmol) and 2-(di-*tert*-butylphosphino)biphenyl (0.65 g, 2.18 mmol) were stirred in anhydrous toluene and reaction was carried out as described in general procedure III to afford **7a** (1.4 g) in 70% yield. ^1^H NMR (500 MHz, CDCl_3_): δ 7.32 (d, *J* = 8.5 Hz, 2H), 7.14 (d, *J* = 9.0 Hz, 2H), 6.89–6.92 (m, 4H), 6.64 (dd, *J* = 10.6, 17.5 Hz, 1H), 5.59 (d, *J* = 17.5 Hz, 1H), 5.09 (d, *J* = 10.9 Hz, 1H), 4.43–4.46 (m, 1H), 3.49–3.54 (m, 2H), 3.11–3.15 (m, 2H), 2.07–2.11 (m, 2H), 1.91–1.96 (m, 2H); LCMS: 364 (M^+^+1).

2-(4-(4-(Trifluoromethoxy)phenoxy)piperidin-1-yl)-5-vinylpyridine **7b:** 2-Bromo-5-vinylpyridine (1.0 g, 5.43 mmol), **6a** (1.70 g, 6.52 mmol), diacetoxypalladium (0.24 g, 1.08 mmol), sodium butan-1-olate (1.25 g, 13.04 mmol) and 2-(di-*tert*-butylphosphino)biphenyl (0.64 g, 2.17 mmol) were stirred in anhydrous toluene and reaction was carried out as described in general procedure III to afford **7b** (1.2 g) in 62.5% yield. ^1^H NMR (400 MHz, CDCl_3_): δ 8.17 (dt, *J* = 0.7, 2.3 Hz, 1H), 7.61 (ddd, *J* = 0.5, 2.5, 8.9 Hz, 1H), 7.09–7.19 (m, 2H), 6.87–6.98 (m, 2H), 6.54–6.71 (m, 2H), 5.57 (dd, *J* = 0.9, 17.6 Hz, 1H), 5.11 (dd, *J* = 0.9, 10.9 Hz, 1H), 4.51 (tt, *J* = 3.6, 7.3 Hz, 1H), 3.92 (ddd, *J* = 3.8, 7.5, 13.2 Hz, 2H), 3.48 (ddd, *J* = 3.6, 8.0, 13.3 Hz, 2H), 1.98–2.10 (m, 2H), 1.79–1.92 (m, 2H); LCMS: 365 (M^+^+1).

1-(2-Fluoro-4-vinylphenyl)-4-(4-(trifluoromethoxy)phenoxy)piperidine **7c:** 1-Bromo-2-fluoro-4-vinylbenzene (2.0 g, 9.95 mmol), **6a** (3.12 g, 11.94 mmol), diacetoxypalladium (0.44 g, 1.99 mmol), sodium butan-1-olate (2.29 g, 23.88 mmol) and 2-(di-*tert*-butylphosphino)biphenyl (1.18 g, 3.98 mmol) were stirred in anhydrous toluene and reaction was carried out as described in general procedure III to afford **7c** (2.1 g) in 55% yield. ^1^H NMR (400 MHz, CDCl_3_): δ 6.88–7.20 (m, 7H), 6.55–6.68 (m, 1H), 5.63 (dd, *J* = 0.8, 17.6 Hz, 1H), 5.19 (d, *J* = 10.8 Hz, 1H), 4.46 (tt, *J* = 3.6, 7.3 Hz, 1H), 3.34 (ddd, *J* = 3.3, 7.6, 11.2 Hz, 2H), 3.01 (ddd, *J* = 3.5, 7.8, 11.6 Hz, 2H), 2.08–2.20 (m, 2H), 1.99 (ddt, *J* = 3.5, 7.3, 16.5 Hz, 2H); LCMS: 382 (M^+^+1).

4-(4-Fluorophenoxy)-1-(4-vinylphenyl)piperidine **7d:** 1-Bromo-4-vinylbenzene (0.56 g, 3.06 mmol), **6b** (0.71 g, 3.67 mmol), diacetoxypalladium (0.13 g, 0.61 mmol), sodium butan-1-olate (0.70 g, 7.34 mmol) and 2-(di-*tert*-butylphosphino)biphenyl (0.36 g, 1.22 mmol) were stirred in anhydrous toluene and reaction was carried out as described in general procedure III to afford **7d** (0.5 g) in 55% yield. ^1^H NMR (400 MHz, CDCl_3_): δ 7.23–7.38 (m, 2H), 6.82–7.03 (m, 6H), 6.64 (dd, *J* = 17.6, 10.9 Hz, 1H), 5.59 (dt, *J* = 17.6, 0.8 Hz, 1H), 5.09 (dt, *J* = 10.9, 0.9 Hz, 1H), 4.38 (tt, *J* = 7.5, 3.7 Hz, 1H), 3.53 (ddd, *J* = 11.6, 7.2, 3.7 Hz, 2H), 3.11 (ddd, *J* = 12.2, 8.2, 3.5 Hz, 2H), 2.08 (ddd, *J* = 13.8, 7.8, 3.8 Hz, 2H), 1.91 (dtd, *J* = 12.0, 7.8, 3.6 Hz, 2H); LCMS: 298 (M^+^+1).

2-(4-(4-Fluorophenoxy)piperidin-1-yl)-5-vinylpyridine **7e:** 2-Bromo-5-vinylpyridine (0.2 g, 1.08 mmol), **6b** (0.25 g, 1.30 mmol), diacetoxypalladium (0.04 g, 0.21 mmol), sodium butan-1-olate (0.25 g, 2.61 mmol) and 2-(di-*tert*-butylphosphino)biphenyl (0.13 g, 0.43 mmol) were stirred in anhydrous toluene and reaction was carried out as described in general procedure III to afford **7e** (0.18 g) in 58% yield. ^1^H NMR (400 MHz, CDCl_3_): δ 8.16 (dd, *J* = 2.4, 0.7 Hz, 1H), 7.60 (dd, *J* = 8.8, 2.5 Hz, 1H), 6.94–7.01 (m, 2H), 6.85–6.91 (m, 2H), 6.54–6.70 (m, 2H), 5.56 (dt, *J* = 17.6, 0.7 Hz, 1H), 5.11 (dt, *J* = 10.9, 0.7 Hz, 1H), 4.44 (tt, *J* = 7.4, 3.6 Hz, 1H), 3.92 (ddd, *J* = 13.2, 7.3, 3.8 Hz, 2H), 3.45 (ddd, *J* = 13.2, 8.1, 3.6 Hz, 2H), 1.97–2.09 (m, 2H), 1.77–1.90 (m, 2H); LCMS: 299 (M^+^+1).

1-(2-Fluoro-4-vinylphenyl)-4-(4-fluorophenoxy)piperidine **7f:** 1-Bromo-2-fluoro-4-vinylbenzene (0.35 g, 1.74 mmol), **6b** (0.40 g, 2.08 mmol), diacetoxypalladium (0.07 g, 0.34 mmol), sodium butan-1-olate (0.40 g, 4.18 mmol) and 2-(di-*tert*-butylphosphino)biphenyl (0.20 g, 0.69 mmol) were stirred in anhydrous toluene and reaction was carried out as described in general procedure III to afford **7f** (0.27 g) in 50% yield. ^1^H NMR (400 MHz, CDCl_3_): δ 7.04–7.14 (m, 2H), 6.56–6.66 (m, 1H), 5.62 (d, *J* = 17.6 Hz, 1H), 5.17 (d, *J* = 8.8, Hz, 1H), 4.38 (tt, *J* = 7.4, 3.7 Hz, 1H), 3.35 (ddd, *J* = 11.4, 7.3, 3.5 Hz, 2H), 2.99 (ddd, *J* = 11.6, 7.9, 3.5 Hz, 2H), 2.07–2.15 (m, 2H), 1.92–2.01 (m, 2H); LCMS: 316 (M^+^+1).

### General Procedure IV, for Preparation of 9a**–**f

At room temperature, with vigorous stirring, a solution of Et_3_N (1.2 mmol) in anhydrous CHCl_3_ was slowly added to a solution of olefin (1.0 mmol) and N-hydroxy-5-nitrofuran-2-carbimidoyl chloride **8** (1.2 mmol) in anhydrous CHCl_3_. The reaction mixture was stirred at room temperature for 2 h then diluted with excess CHCl_3_, washed with water, dried over anhydrous Na_2_SO_4_, concentrated under reduced pressure and purified by flash chromatography to afford compounds **9a–i** in 50–68% yields.

3-(5-Nitrofuran-2-yl)-5-(4-(4-(4-(trifluoromethoxy)phenoxy)piperidin-1-yl)phenyl)-4,5-dihydroisoxazole **9a:** To a solution of **7a** (2.6 g, 7.16 mmol) and **8** (1.63 g, 8.59 mmol) in anhydrous CHCl_3_ (20 mL) was added Et_3_N (1.19 mL, 8.59 mmol) in CHCl_3_ (5 mL) and the reaction continued as described in general procedure IV to afford 2.4 g of **9a** in 65% yield. ^1^H NMR (400 MHz, CDCl_3_): δ 7.39 (d, *J* = 3.9 Hz, 1H), 7.26 (s, 2H), 7.10–7.18 (m, 2H), 7.04 (d, *J* = 3.8 Hz, 1H), 6.86–7.00 (m, 4H), 5.75 (dd, *J* = 8.9, 11.1 Hz, 1H), 4.45 (dt, *J* = 3.7, 7.4 Hz, 1H), 3.75 (dd, *J* = 11.1, 17.2 Hz, 1H), 3.52 (ddd, *J* = 3.7, 7.5, 11.7 Hz, 2H), 3.40 (dd, *J* = 8.9, 17.2 Hz, 1H), 3.15 (ddd, *J* = 3.5, 7.9, 12.1 Hz, 2H), 2.04–2.14 (m, 2H), 1.87–1.99 (m, 2H); ^13^C NMR (101 MHz, CDCl_3_): δ 155.77, 151.49, 147.84, 147.55, 142.84, 131.86, 129.35, 127.21, 122.98, 122.51, 121.82, 116.84, 116.30, 113.08, 112.39, 84.10, 72.70, 46.20, 41.15, 30.20; HRMS m/z [M+H]^+^ calculated for C_25_H_22_F_3_N_3_O_6_∶518.1539; found: 518.1533; Anal. Calculated for C_25_H_22_F_3_N_3_O_6_: C, 58.03; H, 4.29; N, 8.12; F, 11.01. Found: C, 58.22; H, 4.11; N, 8.24; F, 10.85.

3-(5-Nitrofuran-2-yl)-5-(6-(4-(4-(trifluoromethoxy)phenoxy)piperidin-1-yl)pyridin-3-yl)-4,5-dihydroisoxazole **9b:** To a solution of **7b** (1.2 g, 3.29 mmol) and **8** (0.75 g, 3.95 mmol) in anhydrous CHCl_3_ (15 mL) was added Et_3_N (0.55 mL, 3.95 mmol) in CHCl_3_ (2 mL) and the reaction continued as described in general procedure IV to afford 1.1 g of **9b** in 66% yield. ^1^H NMR (400 MHz, CDCl_3_): δ 8.16 (d, *J* = 2.5 Hz, 1H), 7.49 (dd, *J* = 2.6, 8.9 Hz, 1H), 7.42 (d, *J* = 3.8 Hz, 1H), 7.11–7.20 (m, 2H), 7.06 (d, *J* = 3.9 Hz, 1H), 6.87–6.98 (m, 2H), 6.72 (d, *J* = 8.9 Hz, 1H), 5.74 (dd, *J* = 9.1, 11.0 Hz, 1H), 4.53 (tt, *J* = 3.5, 7.2 Hz, 1H), 3.92 (ddd, *J* = 3.8, 7.6, 12.0 Hz, 2H), 3.77 (dd, *J* = 11.1, 17.3 Hz, 1H), 3.52 (ddt, *J* = 3.3, 7.2, 13.8 Hz, 2H), 3.39 (dd, *J* = 9.1, 17.3 Hz, 1H), 2.04 (ddt, *J* = 3.6, 7.3, 11.5 Hz, 2H), 1.79–1.92 (m, 2H); ^13^C NMR (101 MHz, CDCl_3_): δ 159.38, 155.77, 147.93, 147.30, 146.64, 135.51, 122.61, 116.86, 113.06, 112.52, 107.11, 82.30, 73.03, 42.23, 40.75, 30.06; HRMS m/z [M+H]^+^ calculated for C_24_H_21_F_3_N_4_O_6_∶519.1491; found: 519.1514.

5-(3-Fluoro-4-(4-(4-(trifluoromethoxy)phenoxy)piperidin-1-yl)phenyl)-3-(5-nitrofuran-2-yl)-4,5-dihydroisoxazole **9c:** To a solution of **7c** (1.8 g, 4.72 mmol) and **8** (0.98 g, 5.19 mmol) in anhydrous CHCl_3_ (20 mL) was added Et_3_N (0.72 mL, 5.19 mmol) in CHCl_3_ (3 mL) and the reaction continued as described in general procedure IV to afford 1.5 g of **9c** in 60% yield. ^1^H NMR (400 MHz, CDCl_3_): δ 7.40 (d, *J* = 3.8 Hz, 1H), 6.87–7.19 (m, 8H), 5.76 (dd, *J* = 8.4, 11.1 Hz, 1H), 4.45 (tt, *J* = 3.6, 7.1 Hz, 1H), 3.79 (dd, *J* = 11.2, 17.2 Hz, 1H), 3.29–3.44 (m, 3H), 3.02 (t, *J* = 9.7 Hz, 2H), 2.08–2.18 (m, 2H), 1.94–2.03 (m, 2H); ^13^C NMR (101 MHz, CDCl_3_): δ 155.81, 147.74, 147.16, 142.82, 140.77, 133.40, 122.50, 122.07, 119.57, 116.87, 113.96, 113.74, 113.04, 112.66, 83.09, 72.42, 47.64, 41.50, 30.74; HRMS m/z [M+H]^+^ calculated for C_25_H_21_F_4_N_3_O_6_∶536.1444; found: 536.1439.

5-(4-(4-(4-Fluorophenoxy)piperidin-1-yl)phenyl)-3-(5-nitrofuran-2-yl)-4,5-dihydroisoxazole **9d:** To a solution of **7d** (0.23 g, 0.77 mmol) and **8** (0.17 g, 0.92 mmol) in anhydrous CHCl_3_ (5 mL) was added Et_3_N (0.12 mL, 0.92 mmol) in CHCl_3_ (1 mL) and the reaction continued as described in general procedure IV to afford 0.19 g of **9d** in 59% yield. ^1^H NMR (400 MHz, CDCl_3_): δ 7.39 (d, *J* = 3.8 Hz, 1H), 7.20–7.29 (m, 2H), 7.02–7.05 (m, 1H), 6.93–7.00 (m, 4H), 6.85–6.90 (m, 2H), 5.75 (dd, *J* = 11.1, 8.9 Hz, 1H), 4.39 (tt, *J* = 7.4, 3.6 Hz, 1H), 3.74 (dd, *J* = 17.2, 11.1 Hz, 1H), 3.52 (ddd, *J* = 11.8, 7.4, 3.7 Hz, 2H), 3.40 (dd, *J* = 17.2, 9.0 Hz, 1H), 3.13 (ddd, *J* = 12.2, 8.1, 3.5 Hz, 2H), 2.01–2.13 (m, 2H), 1.84–1.97 (m, 2H); ^13^C NMR (101 MHz, CDCl_3_): δ 158.61, 156.24, 153.28, 152.11, 151.54, 147.84, 147.56, 129.27, 127.21, 117.56, 116.28, 116.05, 115.82, 113.08, 112.38, 84.12, 73.18, 46.25, 41.14, 30.29; HRMS m/z [M+H]^+^ calculated for C_24_H_22_FN_3_O_5_∶416.1621; found: 452.1622.

5-(6-(4-(4-Fluorophenoxy)piperidin-1-yl)pyridin-3-yl)-3-(5-nitrofuran-2-yl)-4,5-dihydroisoxazole **9e:** To a solution of **7e** (0.15 g, 0.50 mmol) and **8** (0.11 g, 0.60 mmol) in anhydrous CHCl_3_ (5 mL) was added Et_3_N (84 uL, 0.60 mmol) in CHCl_3_ (1 mL) and the reaction continued as described in general procedure IV to afford 0.14 g of **9e** in 64% yield. ^1^H NMR (400 MHz, CDCl_3_): δ 8.16 (dt, *J* = 2.4, 0.7 Hz, 1H), 7.45–7.50 (m, 1H), 7.38–7.42 (m, 1H), 7.03–7.06 (m, 1H), 6.94–7.01 (m, 2H), 6.85–6.91 (m, 2H), 6.71 (dd, *J* = 8.9, 0.8 Hz, 1H), 5.72 (dd, *J* = 11.0, 9.1 Hz, 1H), 4.45 (tt, *J* = 7.3, 3.6 Hz, 1H), 3.92 (ddd, *J* = 13.5, 7.4, 3.7 Hz, 2H), 3.75 (dd, *J* = 17.3, 11.0 Hz, 1H), 3.32–3.55 (m, 3H), 1.96–2.07 (m, 2H), 1.77–1.90 (m, 2H); ^13^C NMR (101 MHz, CDCl_3_): δ 159.41, 158.64, 156.26, 153.27, 147.93, 147.30, 146.63, 135.49, 122.62, 117.59, 116.07, 115.84, 113.05, 112.52, 107.11, 82.31, 73.51, 42.29, 40.74, 30.15; HRMS m/z [M+H]^+^ calculated for C_23_H_21_FN_4_O_5_∶453.1574; found: 453.1589.

5-(3-Fluoro-4-(4-(4-fluorophenoxy)piperidin-1-yl)phenyl)-3-(5-nitrofuran-2-yl)-4,5-dihydroisoxazole **9f:** To a solution of **7f** (0.08 g, 0.25 mmol) and **8** (0.05 g, 0.30 mmol) in anhydrous CHCl_3_ (2 mL) was added Et_3_N (42 uL, 0.30 mmol) in CHCl_3_ (0.5 mL) and the reaction continued as described in general procedure IV to afford 0.06 g of **9f** in 55% yield. ^1^H NMR (400 MHz, CDCl_3_): δ 7.39 (dd, *J* = 3.9, 0.6 Hz, 1H), 7.02–7.08 (m, 3H), 6.94–7.01 (m, 3H), 6.86–6.91 (m, 2H), 5.75 (dd, *J* = 11.1, 8.4 Hz, 1H), 4.38 (tt, *J* = 7.2, 3.6 Hz, 1H), 3.79 (dd, *J* = 17.2, 11.2 Hz, 1H), 3.29–3.44 (m, 3H), 2.94–3.05 (m, 2H), 2.05–2.17 (m, 2H), 1.90–2.02 (m, 2H); ^13^C NMR (101 MHz, CDCl_3_): δ 158.62, 156.86, 156.24, 154.40, 153.32, 147.73, 147.19, 140.84, 133.33, 122.04, 119.44, 117.60, 116.04, 115.81, 113.95, 113.73, 113.01, 112.60, 83.10, 72.92, 47.70, 41.51, 30.84, 13.61; HRMS m/z [M+H]^+^ calculated for C_24_H_21_F_2_N_3_O_5_∶470.1527; found: 470.1533.

### MIC Determination

Minimum inhibitory concentrations (MICs) were determined according to Clinical Laboratory Standards Institute (CLSI), using microbroth dilution method in 96-well plates and read by visual inspection [21]. The lowest concentration of drug that prevented visible growth was recorded as the MIC. Liquid MICs for indicated mycobacterium were determined in Middlebrook 7H9 broth supplemented with 10% albumin–dextrose complex and 0.05% (v/v) Tween 80 after 3 days (*M. abscessus, M. fortuitum*), or 7 days (*M. avium, M. kansasii, M. tuberculosis*) unless otherwise indicated. *M. ulcerans* MICs were performed by the agar proportion method and results recorded after 4 weeks of incubation.

### Minimum Bactericidal Concentration Determination

Liquid MICs were performed as described above with the additional step of determining the number of bacteria added to each well. Serial dilutions of bacteria from the MIC plates were prepared and spread on Middlebrook 7H11 agar supplemented with 10% oleic acid–albumin–dextrose complex. After 4 weeks of incubation at 37°C, colony forming units (CFUs) were counted and the input calculated. Following 7 days of incubation, the contents of individual MIC plate wells were resuspended and spread on drug-free 7H11 agar plates. Plates were incubated at 37°C for 4 weeks prior to enumeration of viable colonies. The MBC was recorded as the lowest treatment concentration that killed >99.9% of input bacteria.

### Cytotoxicity

*In vitro* cytotoxicity was assessed using Vero cells (kidney epithelial cells; ATCC CCL-81) essentially as described previously, except that detection was performed using CellTiter-Glo® Luminescent Cell Viability (Promega) [22]. The concentration of treatments that produced a 50% decrease in viability (IC_50_ values) were computed using nonlinear regression assisted equation of log [inhibitor] vs. Response-variable slope (four parameters) symmetrical, in GraphPad prism software.

### Post Antibiotic Effect (PAE)

PAE studies were performed essentially as described previously [21]. To facilitate sample handling, *M. bovis* strain BCG Pasteur was used as a surrogate for *M. tuberculosis* [21]. Briefly, a mid-log phase culture recovered from frozen stock was diluted to an OD_600_ of ∼0.001 and allowed to grow to early log-phase (OD_600_ 0.2). For each experimental condition, 50 mL of cells were exposed to **9a** (at 0.1, 1.0, or 10.0 µg/mL) or isoniazid (at 0.05, 0.5, or 5.0 µg/mL) for 2 hours. Drugs were subsequently removed by washing twice in pre-warmed Middlebrook 7H9 medium and treated cells resuspended in pre-warmed medium. OD_600_ was determined for each culture immediately following resuspension and every 24 hours thereafter until reaching growth saturation. The difference between the time required for treated cells to reach 50% of the maximum OD_600_ of the untreated culture and for the untreated culture to reach this density was calculated as the PAE. Average and standard deviation were calculated based on results of two biologically independent experiments.

### *In vitro* Activity against NRP Bacteria

To evaluate the activity of compounds against dormant TB bacilli, two *in vitro* models were used, mimicking oxygen and nutrient starvation. These experiments were performed using *M. tuberculosis* strain H37Rv as described previously, with no modifications [23], [24]. Briefly, hypoxic cultures were exposed to 10 µg/mL of indicated treatments for 4 days prior to cfu enumeration. For nutrient starvation experiments, aliquots of six-week starved cultures (in PBS) were exposed to indicated treatments for one week prior to cfu enumeration.

### Checkerboard Synergy Assays

Whole cell *in vitro* synergy assays were performed using *M. tuberculosis* strain H37Rv. In a 96-well assay plate, two fold serial dilutions of Drug A (ethambutol, isoniazid, linezolid, PA-824, rifampin, or streptomycin) were prepared in 100 µl of Middlebrook 7H9 media (highest and lowest concentrations in rows A and G, respectively, with no drug in row H). Using a single dip with a 200ss pintool, 0.2 µl of drug B (**9a**) was transferred to the assay plate columns 1 to 11 of the assay plate, with drug-free DMSO transferred to column 12. To each well of the assay plate 100 µl of mid-log phase bacteria (diluted to OD_600_ of 0.01) was added, and plates incubated for 7 days prior to reading MICs by visual inspection. Fractional inhibitory concentration index (FICI) scores were calculated using the formula [MIC drug B in presence of Drug A]/[MIC of drug B)+[MIC of drug A in the presence of drug B]/[MIC of drug A]. FICI scores were interpreted as follows: synergy (≤0.5), indifference (>0.5–4.0), or antagonism (>4.0) [25]. For each drug combination, FICI ranges were reported from two biologically independent experiments.

### *In vitro* Drug Resistance Frequency

Resistance frequency determination and spontaneous mutants selection was performed by plating *M. tuberculosis* H37Rv in the presence of increasing super-MIC concentrations of compound as described previously [21]. Where indicated, liquid cultures were exposed to a sub-inhibitory concentration of **9a** prior to selection on agar.

### Pharmacokinetic Analyses

Maximum solubility, metabolic stability, and plasma protein binding assays were performed and analyzed as described previously [22]. For *in vivo* pharmacokinetic studies, catheterized male Sprague-Dawley rats were obtained from Harlan Bioscience (Indianapolis, IN) and maintained as previously described [22]. Test compounds were dissolved in 30% DMSO, 30% propylene glycol, 20% PEG 3000 and 20% saline and administered intravenously (IV) at 10 mg/kg or were dissolved in PEG-3000:Water (60∶40) and dosed by oral gavage at 100 mg/kg. Blood sample collection, processing, and analysis were performed exactly as described previously [22]. The pharmacokinetic parameters maximum plasma concentration (C_max_), minimum plasma concentration within 24 hours after dosing (C_min,24****h_), systematic exposure (AUC_0-∞_), half-life (t_1/2_), clearance (CL), volume of distribution (Vd), fraction excreted unchanged in urine (f_e_), and oral bioavailability (F) were determined by standard non-compartmental analysis using the software package Phoenix WinNonlin 6.2 (Pharsight, Mountain View, CA).

### *In vivo* Efficacy

Efficacy of **9a** in an acute tuberculosis infection mouse model was performed as described previously [26], [27], [28]. Briefly, 8 week female GKO mice (C57BL/6-Ifngtm1 Ts, from Jackson Laboratories) were infected with a low dose aerosol inoculum (∼100 CFU’s per mouse) of *M. tuberculosis* Erdman, transformed with pFCA-LuxAB. Isoniazid was dissolved in distilled water (DI). Treatment start was 14 days after infection. **9a** was assessed in various formulations and therefore prepared using several different carriers (either 5% methylcellulose in DI-H_2_O, 30% captisol in DI-H_2_O, 10% vitamin E TPGS in DI-H_2_O, 0.5% Tween 80 in DI-H_2_O, 20% non-functionalized cyclodextrin (2-hydroxypropyl-β-cyclodextrin) in DI-H_2_O, or cold PEG (50∶35∶15 H_2_O:PEG300:PG)). Groups of 5 mice per group received **9a** (300 mg/kg), isoniazid (25 mg/kg), or no treatment (untreated control group) for 9 consecutive days by oral gavage prior to sacrifice. Lungs and spleens from drug treated and control groups were homogenized and bacterial loads determined by CFU enumeration to determine bacterial loads before and after the 9 day treatment period, as described previously [27]. Reduction in bacterial loads of treatment groups compared to the untreated control group was recorded as (Log_10_) reduction. For statistical analysis the CFU were converted to logarithms, which were then evaluated by a one-way analysis of variance and two-way analysis of variance, followed by a multiple comparison analysis of variance by a one-way Tukey test and Dunnett test (SAS Software program, Research Triangle Park, NC). Differences were considered significant at the 95% level of confidence.

### Ethics Statement

The pharmacokinetic study protocol was approved by Institutional Animal Care and Use Committee of the University of Tennessee Health Science Center (Protocol number 1463). The *in vivo* efficacy study protocol was approved by Colorado State University Institutional Animal Care and Use Committee (Protocol number 13-4263A).

## Results and Discussion

### Synthesis of an Expanded Set of Pentacyclic Nitrofurans

**Figure 2:**
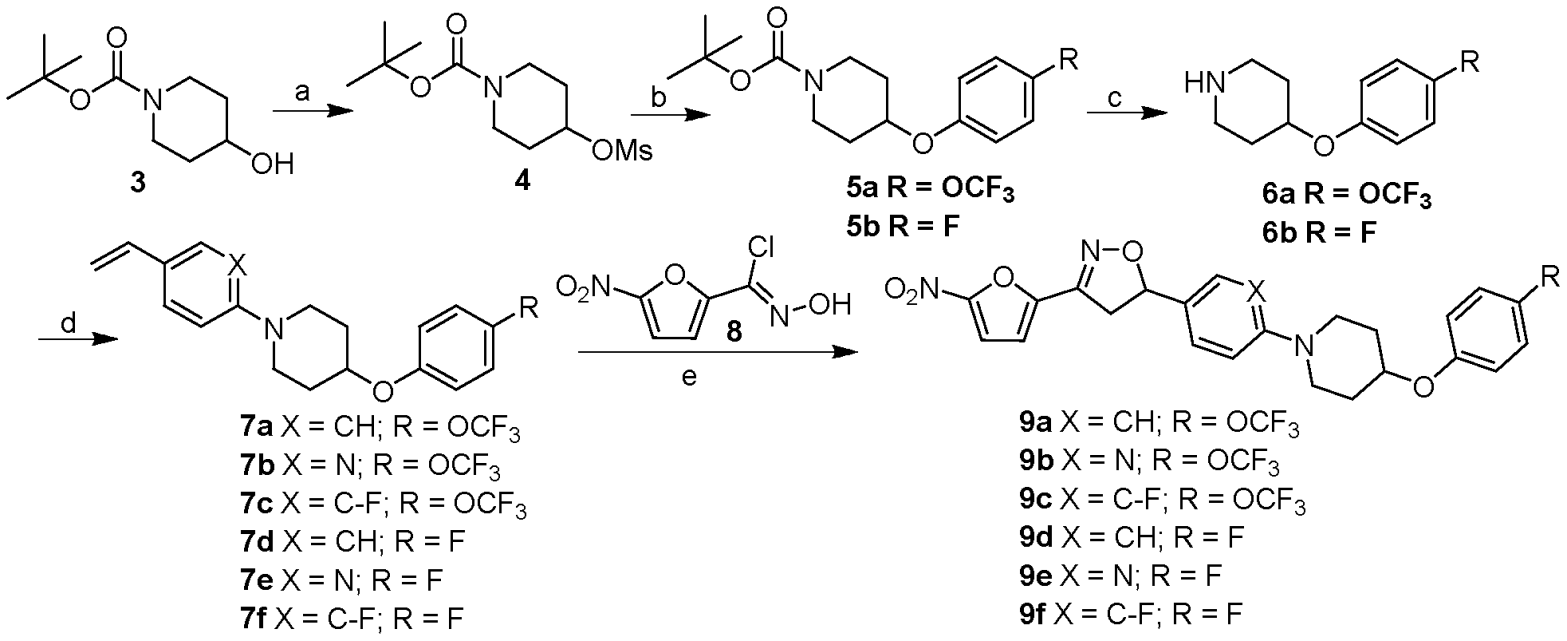
Synthesis of pentacyclic nitrofurans. Reagents and conditions: a) MsCl, Et_3_N, CH_2_Cl_2_, RT, 2 h, 94%; b) substituted phenol, *n*Bu_4_NCl, H_2_O, 100°C, 12 h, 86–90%; c) TFA, CH_2_Cl_2_, RT, 1 h, 92–95%; d) aryl bromide, 2-(di-*tert*-butylphosphino)biphenyl, NaO*t*Bu, Pd(OAc)_2_, toluene, 100°C, 3 h, 50–70%; e) **8**, Et_3_N, CHCl_3_, RT, 3 h, 50–68%.

The design of the target series of new pentacyclic nitrofurans is shown in Figure 1. The nitrofuranyl and isoxazoline rings of our previous lead compounds **1**, **2** and **9a** were kept intact to maintain antitubercular potency, whereas the outer side chain (C-E rings) of the pentacyclic system were modified [18], [22]. Within this general scaffold, modifications were targeted to the C and E rings with substitutions aimed at blocking sites of potential metabolism and increasing solubility. The target compounds **9a–f** were synthesized in a five step sequence starting from 1-Boc-4-hydroxypiperidine **3** as shown in Figure 2. Accordingly, **3** was mesylated by treating with methanesulfonyl chloride and triethylamine in dichloromethane at 0°C for 1 h to yield **4** in 93% yield which upon treatment with appropriate substituted E-ring phenols in presence of potassium carbonate and tetra *n*-butyl ammonium chloride in water at reflux afforded ethers **5a,b** in 86–90% yields. Boc deprotection of **5a,b** with trifluoroacetic acid in dichloromethane at room temperature produced the key intermediate amines **6a,b**. The aryl amination reactions were carried out under Buchwald conditions by using appropriate C ring aryl bromides and secondary amines **6a,b** in presence of palladium catalyst and sodium *tert*-butoxide in toluene at 100°C to afford **7a–f** in 50–70% yields [22]. Finally, the isoxazoline ring was constructed by treating the olefins with nitrofuranyl chloroxime **8** [29] in the presence of triethylamine in chloroform at room temperature to give compounds **9a–f** in 50–68% yields.

### Antituberculosis Activity and *in vitro* Metabolic Profile

All the compounds in the series maintained excellent potency against *M. tuberculosis* (MIC 0.001–0.02 µg/mL, corresponding to 1.9–46 nM), as shown in Table 1, which was well within their C_max_ range. Mammalian cytotoxicity assays and calculation of subsequent therapeutic indices revealed very favorable selectivity, which ranged from 150–2500. Solubility of the series was low, however, with modifications yielding a ∼10 fold drop in solubility compared to our earlier series (**2**). Plasma protein binding remained very high (>96%) for all analogs. Compounds with a 4-fluorophenyl E ring (**9d–f**) showed a slight improvement in microsomal stability (t_½_ of 1.4–1.9 h. compared to 1.1 h for **2**). The 4-trifluoromethoxyphenyl E ring series (**9a–c**), however, demonstrated much improved metabolic stability (t_½_ of 5–7 hours). Modification to the C ring (pyridyl in **9b** and **9e**, fluorophenyl in **9c** and **9f**) had negligible effects on overall properties irrespective of E ring substitution.

**Table 1:** *In vitro* antitubercular and in vitro metabolic profile.

Compound	a *M. tb.* MIC(µg/mL)	bCytotoxicityIC_50_ (µg/mL)	cSelective Index(Cytox/MIC)	dSolubility (pH 7.4)µg/mL	eMicrosomal stability t_1/2_(mouse) (h)	fPlasma Proteinbinding (%)
**2**	0.006	2.9^g^	483	9.9	1.06^g^	96.4^g^
**9a**	0.024	19.4	808	2.7^h^	5.06	99.9^h^
**9b**	0.006	4.0	667	1.6	6.91	99.9
**9c**	0.001	21.4	21400	1.9	7.04	97.6
**9d**	0.012	14.7	1225	0.7	1.43	99.1
**9e**	0.002	7.5	3750	2.2	1.93	99.8
**9f**	0.012	8.0	667	0.8	1.74	99.8
**OPC-67683**	0.01	107.1	10710	1.22	27.25	99.8
**PA-824**	0.39	69.4	178	8.5	8.12	92.9

Structures, inhibitory activity, and *in vitro* PK properties of experimental and reference nitrofurans.

a The lowest concentration of drug that prevented visible growth.

b Cytotoxicity against Vero epithelial cells using MTT assay.

c Selectivity for tuberculosis.

d Maximum solubility calculated by µSol Evolution Software.

e Metabolic stability performed with mouse liver microsomes.

f Plasma protein binding assays performed using mouse plasma.

g Indicates values reported in reference.

h Indicates values reported in reference.

### Spectrum of Antimicrobial Activity

Compounds **9a–f** were weakly active or inactive against a panel of gram-positive and gram-negative pathogens (Table S1) except for **9f**, which had moderate activity against most gram-positive pathogens and an efflux pump deficient strain of *E. coli.* This activity is consistent to that observed for other similar nitrofurans we generated in prior series [21], [22]. Compound **9a** was further tested against non-tuberculosis disease causing mycobacteria (Table 2). Compound **9a** was moderately active against non-tuberculosis mycobacteria (NTM) *M. abscessus*, *M. kansasii* and *M. fortuitum* and highly active against *M. ulcerans* with an MIC lower than the plasma concentrations maintained over 24 hours after administration, with a C_max_ of 0.85 µg/mL and a C_min,24****h_ of 0.17 µg/mL. PA-824 was moderately active against *M. kansasii* and *M. ulcerans* but inactive against other NTM species tested, in agreement with previously reported studies [30], [31]. The activity for **9a** against *M. abscessus* is notable since this difficult to treat mycobacterium is intrinsically resistant to most anti-mycobacterial agents [32]. These differences in the spectrum of activity between the nitrofurans and the nitroimidazole PA-824 is consistent with differences in the redox potential for these two compound classes, with the lower redox potential of nitroimidazoles requiring specific enzymes for activation, whilst nitrofurans have a higher redox potential and may be more easily activated by a wider spectrum of enzymes [17], [33].

**Table 2:** Susceptibility of non-tuberculosis mycobacteria.

	Minimum Inhibitory Concentration (µg/mL)				
Compound	a *M. abscessus*	a *M. avium*	a *M. kansasii*	a *M. fortuitum*	b *M. ulcerans*
**9a**	12.50	200	3.13–6.25	6.25–12.50	0.02–0.05
Amikacin	3.13–6.25	12.5	6.25–12.5	0.78	ND
Rifampin	50.00	6.3	0.39	1.56	0.1–0.4
PA-824	>200	>200	3.1–12.5	>200	3.1–25

Susceptibility of non-tuberculosis mycobacteria to nitrofurans and reference drugs.

a MICs determined by macrobroth dilution and visual inspection of plates.

b MIC determined by agar proportion method. Values reported are the range of at least two biologically independent experiments. Species tested included *M. abscessus* (ATCC 19977); *M. avium* (ATCC 25291); *M. kansasii* (ATCC 12478); *M. fortuitum* (ATCC 6841); and *M. ulcerans* (ATCC 35840).

### *In vivo* Pharmacokinetic Profiling

The pharmacokinetic profiles of **9b–c** were evaluated in rats and compared to the published data for **9a** using similar formulations (Table 3). After intravenous administration of 10 mg/kg **9b–c**, compounds showed biexponential concentration-time profiles similar to **9a**, but elimination half-lives (t½ 2.5–5.6 h) significantly lower than **9a** (t½ 10.3 h). The shorter half-lives are a consequence of a reduced volume of distribution for compounds **9b–c** (Vd 1.53–1.69 L/kg) compared to **9a** (Vd 6.72 L/kg), while clearance (CL) remained similar for all three compounds. The fraction of dose excreted unchanged by the kidneys (fe) was negligible for all compounds. Their relatively large volumes of distribution and non-renal elimination are probably a reflection of their high lipophilicity (clogP 4.6–5.8). The oral bioavailability of compounds **9b** and **9c** was poor (F 8.0%, 0.83%), possibly a reflection of poor solubility as had already been noted for **9a** which also has limited oral bioavailability (F 4.6%).Compounds **9a** and **9b** were non-toxic by intravenous and oral dosing in rats. Central nervous system toxicity (seizures) was observed for compound **9c** in both administration modes. Compound **9a** was therefore advanced for *in vivo* efficacy testing due to its longer *in vivo* half-life and higher volume of distribution as compared to **9b**.

**Table 3:** *In vivo* pharmacokinetic parameters.

	After IV administration of 10 mg/kg				After oral administration of 100 mg/kg			
Compound	t_½_ (h)	CL (L/h/kg)	Vd (L/kg)	fe (%)	C_max_ (mg/L)	C_min,24 h_ (mg/L)	AUC_0-∞_ (mg h/L)	F (%)
**9a** ^Ref^	10.3 (1.4)	0.46 (0.07)	6.72 (1.10)	0.04 (0.01)	0.85 (0.80)	0.17 (0.16)	10.2 (9.1)	4.56 (4.06)
**9b**	2.48 (0.62)	0.42 (0.15)	1.53 (0.36)	0.01 (0.00)	2.31 (0.62)	0.11 (0.11)	15.1 (7.1)	8.00 (3.93)
**9c**	5.60 (0.45)	0.41 (0.06)	1.69 (0.27)	0.02 (0.00)	0.15 (0.06)	0.02 (0.02)	1.61 (0.83)	0.83 (0.57)

Pharmacokinetic analysis of experimental compounds in rats.

Values represent means (% coefficient of variation).

Abbreviations: t_½_: half life; CL: clearance; Vd: volume of distribution; fe: fraction excreted unchanged in urine; C_max_: maximum plasma concentration; C_min,24****h_: minimum plasma concentration within 24 hours after dosing; AUC_0-∞_: systematic exposure; F: oral bioavailability.

### *In vivo* Efficacy in a Mouse Model of Acute Tuberculosis Infection

**Figure 3:**
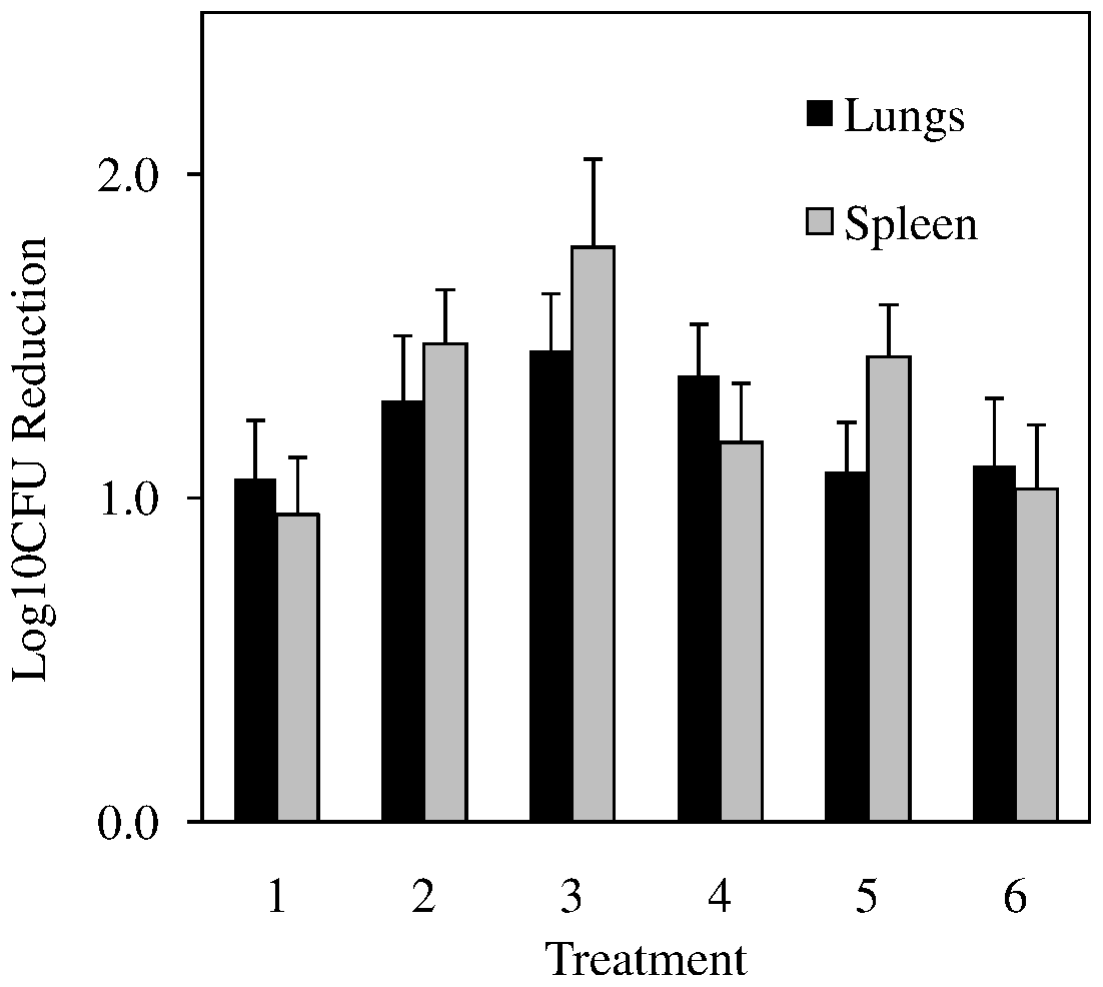
Murine model of acute tuberculosis infection. Log_10_ reduction provided by compound **9a** in lungs (black bars) and spleen (grey bars) after 9 days of daily oral administration of 300 mg/kg was determined by calculating the difference between bacillary loads in organs from the untreated group and **9a** dissolved in (**1**) 0.5% methylcellulose in DI-H_2_O (**2**) 30% captisol in DI-H_2_O (**3**) 10% vitamin E TPGS in DI-H_2_O (**4**) 0.5% Tween 80 in DI-H_2_O (**5**) 20% cyclodextrin in DI-H_2_O or (**6**) cold PEG (50∶35∶15 H_2_O:PEG300:PG). Error bars indicate SEM within treatment groups of 5–7 mice per group.

**9a** was next evaluated for *in vivo* efficacy using a mouse model of acute tuberculosis infection [26]. Given the poor solubility of **9a**, six different formulations were employed in the acute GKO mouse infection model. In each formulation, **9a** was well tolerated with daily oral administration at 300 mg/kg and 9 days of treatment provided a statistically significant reduction (P<0.001 by Tukey Test) in bacillary loads in both the lungs and spleen (Figure 3, Table S2). The differences in efficacy between different **9a** formulation groups were not statistically significant. A control drug isoniazid was included in the experiment (data not shown), which gave an expected a 3.5 and 4.6 log reduction of the bacterial load in lungs and spleen, respectively, versus the untreated controls. Although **9a** did not provide superior protection compared to rapid acting control isoniazid, the reduction it produced in lungs was 91–97% in 9 days. This marks a substantial improvement over precursor tetracyclic nitrofuranyl isoxazolines, which were not active *in vivo* [15].

### Post Antibiotic Effect and *in vitro* Synergy with Frontline Agents

Post antibiotic effect (PAE), defined as the duration of growth retardation following the removal of an antibiotic from the environment of the organism, is an important parameter for early consideration of potential dosing and was determined for compound **9a** [**34**]. Pulse exposure to **9a** yielded a dose-responsive PAE, with compound concentrations of 0.01, 1.0, and 10.0 µg/mL yielding PAE’s of 21.7+/−1.2 h, 105.5+/−19.8 h, and 178.4+/−8.8 h, respectively. The PAE produced by treatment with **9a** at 1 µg/mL (105.5 h) was superior to that produced by exposure to 5 µg/mL isoniazid (71.8 h). The long PAE of compound **9a** is highly favorable for the further development of this antitubercular compound series.

An important characteristic for novel tuberculosis therapeutics is compatibility with antitubercular agents in the clinic and late stage development, since combination therapy is applied to reduce the development of acquired drug-resistance. A preliminary investigation of **9a**’s potential for co-administration was, therefore, conducted using the checkerboard approach (Table 4) [35]. In this commonly used approach to access *in vitro* synergy, serial dilutions of two agents are prepared in opposing directions across an MIC assay plate and the fractional inhibitory concentration index (FICI) is calculated using the formula [MIC drug B in presence of Drug A]/[MIC of drug B)+[MIC of drug A in the presence of drug B]/[MIC of drug A]. No synergy or antagonism was seen with frontline drugs ethambutol and isoniazid, nor with linezolid or streptomycin (FICI scores 0.5–4.0). However, **9a** synergized with the frontline treatment rifampin and nitroimidazole PA-824 (FICI <0.5). Interestingly, in the presence of **9a**, MICs of PA-824 and rifampin both decreased ∼10 fold. The favorable synergy predicted for **9a** with rifampin, as well as the lack of unfavorable interactions predicted with other antitubercular drugs, suggests that **9a** may complement current tuberculosis treatment regimes.

**Table 4:** *In vitro* Interactions with Antitubercular Drugs.

Treatment	Drug Class	MIC Alone(µg/mL)	MIC in presence of9a (µg/mL)	9a MIC in combination(µg/mL)	aFICI with 9a	Type ofInteraction
Rifampin	Rifamycin	0.02–0.05	0.003	0.003–0.01	0.38	Synergy
Isoniazid	Other	0.02	0.01–0.02	0.02–0.0008	0.52–2.00	Indifference
Ethambutol	Antimycobacterial	1.6	0.8	0.01	0.75–1.00	Indifference
Linezolid	Ooxazolidinone	1.6	0.8	0.01–0.02	1.00	Indifference
PA-824	Nitroimidazole	0.2–0.8	0.01–0.02	0.003–0.006	0.13–0.28	Synergy
Streptomycin	Aminoglycoside	1.6	0.4–0.8	0.025–0.006	0.75	Indifference

*In* vitro interactions determined by checkerboard assays.

a Ex-vivo synergy assays were performed to determine if **9a** displayed synergy (FICI ≤0.5), indifference (FICI >0.5–4.0) or antagonism (FICI >4.0) with a panel of anti-tuberculosis agents.

### Minimum Bactericidal Concentration Determinations

The minimum bactericidal concentration (MBC) of **9a** was determined (alongside controls); the results are shown in Table 5. The number of viable bacteria in individual wells of MIC plates was enumerated to determine the minimum bactericidal concentration (MBC), defined as the treatment concentration that killed >99.9% of the inoculum. Streptomycin was cidal (MBC/MIC ≤4), as anticipated, whereas all nitroaromatics compounds investigated were static (MBC/MIC >4). These observations are also consistent with nitrofurans being known to be static at low concentrations but cidal at higher concentrations.

**Table 5:** MIC and MBCs for select nitrofurans and controls.

Treatment	MBC	Classification
**2**	–	–
**9a**	3.13–6.25	Static
OPC-67683	>3	Static
PA-824	>6	Static
Streptomycin	3.13–6.25	Cidal

Treatments were considered cidal when the MBC_99.9_ for *M. tuberculosis* H37Rv was less than 4X the MIC.

### Activity against Multiple Models of Tuberculosis

**Figure 4:**
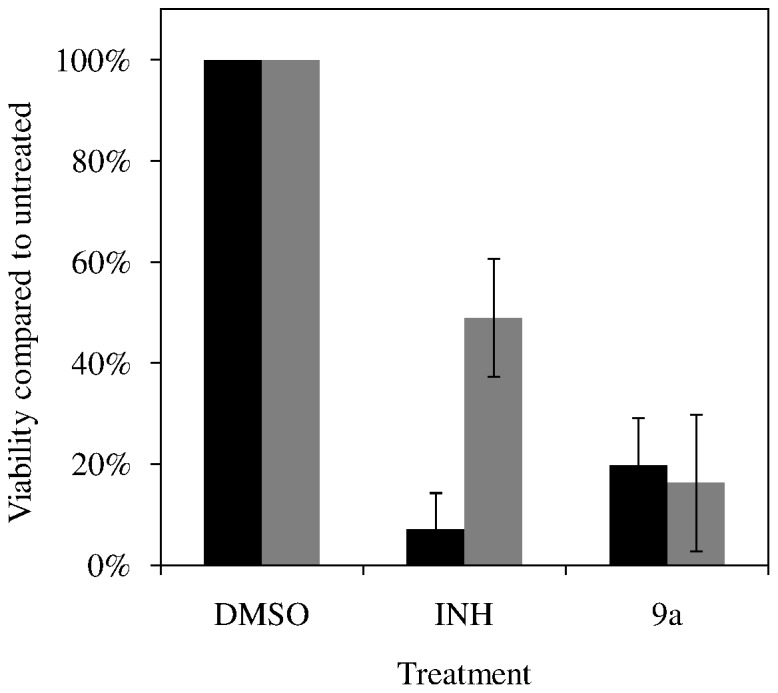
Nutrient starvation model of nonreplicating persisters. The viability of mid-log phase (black bars) or nutrient-starved (gray bars) after exposure to DMSO carrier (1% v/v), 1 µg/mL of isoniazid (INH), or 1 µg/mL of **9a**. Averaged results and SEM from two biologically independent experiments are presented.

Several *in vitro* models have been proposed to mimic the physiological state of persistent tuberculosis infections *in vivo.* The most commonly used model of tuberculosis persistence is based upon the assumption that the host immune system sequesters bacilli into granulomas, where they persist at low oxygen levels. Activity of **9a** was assessed in the Rapid Anaerobic Dormancy (RAD) model which uses rapid oxygen depletion to mimic the Non-Replicating-Persister 2 (NRP-2) conditions described by Voskuil. [24], [36]. As anticipated, NRP-2 bacilli were sensitive to treatment with rifampin but resistant to isoniazid (Table 6). In this *in vitro* model of a dormant tuberculosis, **9a** and PA-824 were both highly active, killing >99.9% of hypoxic bacilli at equivalent concentrations (10 µg/mL). In addition to oxygen deprivation, nutrient starvation is also proposed as an important environmental inducer of persistent tubercular populations. A simple model of starvation-induced persistence reported by Betts *et al.* [23]was adopted to further assess the anti-dormancy activity of nitrofuran **9a** (Figure 4). Mid-log and nutrient-starved cultures were treated with test compound (**9a** or isoniazid at 1 µg/mL) or DMSO drug carrier for 7 days. As anticipated, isoniazid treatment killed >95% of actively growing *M. tuberculosis* culture but was 7 fold less active against nutrient starved bacteria. **9a** produced an 80% reduction in viability of both actively growing and nutrient starved bacilli. The sustained activity of compound **9a** in two separate models of biologically relevant, drug-refractory *M. tuberculosis* populations suggests it may serve as a valuable, treatment-shortening addition to the existing anti-tuberculosis regimen.

**Table 6:** In vitro activity against NRP bacteria grown under hypoxic conditions.

Compound	Concentration	% Growth vs. Control
DMSO (carrier)	1%	100%
Isoniazid	10 µg/mL	29.1–49.1%
Rifampin	10 µg/mL	<0.1%
PA-824	10 µg/mL	<0.1%
**9a**	10 µg/mL	<0.1%

Viability of hypoxic *M. tuberculosis* cultures was assessed using the rapid anaerobic dormancy model.

### Mechanism of Action Studies

All nitroaromatic antibiotics are prodrugs for which bioreduction by the pathogen is required to generate the active antimicrobial metabolites. The bioreduction of PA-824 is well studied and is mediated by the deazaflavin-dependent nitroreductase (Ddn) in *M. tuberculosis*, with cellular bioreduction ultimately producing toxic nitric oxide [37], [38]. The F_420_-dependent glucose 6-phosphate dehydrogenase (FGD1), which is required in the recycling of F_420_ cofactor, is responsible for transferring electrons to Ddn (encoded by Rv3547). Thus mutations in genes encoding Ddn, FGD1 and F_420_ biosynthesis confer resistance to PA-824 and cross resistance to OPC-67683, which is activated in a similar manner [39]. A collection of PA-824 resistant mutants each harboring a mutation in a single gene in the PA-824 activation pathway (*FGD1*, *F_420_*, or *Ddn*) were screened against compound **9a** for cross resistance (Table 7). All these mutants remained sensitive to **9a**, suggesting that **9a** is primarily activated by a different pathway and is unlikely to be affected by primary resistance mechanisms of bicyclic nitroimidazoles.

**Table 7:** Mechanism of activation and primary resistance.

	Genotype			MIC (µg/ml)	
Strain	*FGD1*	*F_420_*	*Ddn*	PA-824	9a
H37Rv-*wt*	**+**	**+**	**+**	0.1	0.025
H37Rv-T3	−	+	+	100	0.1
H37Rv-5A1	+	−	+	50	0.05
H37Rv-14A1	+	+	−	50	0.1

Activity of nitrofurans against *M. tuberculosis* H37Rv mutants deficient in enzymes required for bioreductive activation of PA-824.

To assess *M. tuberculosis’s* potential to become resistant to lead **9a**, the frequency at which spontaneous resistance arises was determined by growing the susceptible *M. tuberculosis* strain H37Rv on agar containing **9a**. Resistant clones arose at a frequency of 3×10^−7^ in the presence of 2–4×MIC of **9a**. Repeated efforts to generate resistant clones at higher concentrations of **9a** were unsuccessful, suggesting the potential for development of resistance is very low, possibly attributable to multiple mechanisms of activation or the induction of alternative modes of action at higher concentrations of compound. This appears consistent with **9a** being bactericidal at higher concentrations. Pre-treatment of cultures with sub-inhibitory concentrations of **9a** permitted selection of resistant colonies on agar plates containing 0.05–0.4 µg/mL of **9a**. All **9a** resistant clones assessed exhibited elevated **9a** MIC (>3.13 µg/mL) but retained sensitivity to ethambutol, linezolid, streptomycin, rifampin, ofloxacin, and isoniazid (Table 8). All but a single **9a** resistant clone remained sensitive to PA-824 and OPC-67683 (Table 8) further supporting the hypothesis that **9a** is primarily activated by a unique biological pathway.

**Table 8:** Cross Resistance Profiles for Compounds 9a Resistant Clones.

Clone #	**Parent**	**1**	**2**	**3**	**4**	**5**	**6**	**7**	**8**	**9**
**Selection Conc. (µg/mL)**	–	0.05	0.05	0.1	0.1	0.2	0.2	0.2	0.4	0.4
**2**	S	**R**	**R**	**R**	**R**	**R**	**R**	**R**	**R**	**R**
**9a**	S	**R**	**R**	**R**	**R**	**R**	**R**	**R**	**R**	**R**
**PA-824**	S	S	S	S	S	**R**	S	S	S	S
**OPC 67683**	S	S	S	S	S	**R**	S	S	S	S

Abbreviations: S, susceptible; **R**, resistant.

## Conclusions

Given the success of nitroaromatic antibiotics in treating anaerobic bacterial infections and the current lack of suitable therapeutics to treat persistent tuberculosis infections, we report here the synthesis and antimicrobial characterization of pentacyclic nitrofuran analogs that incorporate the outer ring elements of OPC-67683. These pentacyclic nitrofurans displayed a favorable *in vitro* selectivity index and selective inhibitory activity against mycobacteria. The MIC of the series lead **9a** (Lee1106) was 46 nM, comparable to that of OPC-67683 (19 nM), and is 20-fold more potent than PA-824 (>1000 nM). Compound **9a** was active in *in vitro* assays against nonreplicating bacteria suggesting its potential for treatment of chronic tuberculosis infections. **9a** exerted a lengthy post antibiotic effect, similar to isoniazid, and was synergistic with the frontline drug rifampin. Mechanism of actions studies showed that the three genes required for activation of PA-824 and OPC-67683 are dispensable for **9a** antitubercular activity, indicating a low potential for cross resistance to other nitroimidazole antitubercular agents. This finding also shows that despite the sharing of common outer ring structural features between **9a** and OPC-67683, the key difference in their nitroaromatic head groups leads to the use of different mechanisms of bioreductive activation. This likely arises from significant differences in the redox potential between nitrofurans and nitroimidazoles, with the latter compounds having a lower redox potential which means that more specific enzymes are required to transfer electrons to the nitro-group. Conversely, the high redox potential for nitrofurans makes these molecules easier to be reduced and different enzymes may be involved [17], [40]. Compound **9a** was well tolerated in a murine model of acute tuberculosis infection, and showed significant *in vivo* efficacy in both lungs and spleens with >90% reduction in bacillary load after 9 days of treatment. In summary, the studies presented here show significant promise for development of the nitrofuran series as antituberculous agents. Further studies to improve metabolic stability, solubility and bioavailability are warranted in order to enhance the *in vivo* efficacy of the series.

## Supporting Information

Table S1 **Spectrum of Antimicrobial Activity.**
(DOCX)

Table S2 **Assessment of Efficacy in an** ***in vivo*** **Murine Model of Acute Tuberculosis Infection.**
(DOCX)
