# Supplementary material for: Pentacyclic Nitrofurans with In Vivo Efficacy and Activity against Nonreplicating Mycobacterium tuberculosis
Source: PLoS One. 2014 Feb 5;9(2):e87909. doi: 10.1371/journal.pone.0087909 (PMC3914891; doi:10.1371/journal.pone.0087909)
Supplement: Table S1 — Spectrum of Antimicrobial Activity. (DOCX) [file pone.0087909.s001.docx]

**Supporting Information – Table S1**

**Pentacyclic nitrofurans with *in vivo* efficacy and activity against latent *Mycobacterium tuberculosis***

Rakesh,^1†^ David F. Bruhn,^1†^ Michael S. Scherman,^2^ Lisa K. Woolhiser,^2^ Dora B. Madhura,^3^ Marcus M. Maddox,^1^ Aman P. Singh,^1,4^ Robin B. Lee,^1^ Julian G. Hurdle,^1‡^ Michael R. McNeil,^2^ Anne J. Lenaerts,^2^ Bernd Meibohm,^3^ Richard E. Lee^1,4*^

^1^ Department of Chemical Biology and Therapeutics, St. Jude Children’s Research Hospital, Memphis, TN, USA

^2^ Mycobacterial Research Laboratories, Department of Microbiology, Colorado State University, Fort Collins, CO, USA

^3^ Department of Pharmaceutical Sciences, College of Pharmacy, University of Tennessee Health Science Center, Memphis, TN, USA

^4^ Biomedical Sciences Program, Graduate Health Sciences, University of Tennessee Health Science Center, Memphis, TN, USA

^‡^ Present address: Department of Biology, University of Texas Arlington, Arlington, TX, USA
* [Richard.Lee@StJude.org](mailto:Richard.Lee@StJude.org) to whom all correspondence should be addressed.

† These authors contributed equally.

**Table S1: Spectrum of Activity**

|  | Minimum Inhibitory Concentration (µg/mL) | | | | | | | | | | |
| --- | --- | --- | --- | --- | --- | --- | --- | --- | --- | --- | --- |
| Compound | *S.a*^a^ | MRSA | *B.s.* | *S. Py.* | *S. Pn.* | *E.f.* | *B.a.* | *B.c.* | *S.m.* | *E.c.* | *E.c. ΔtolC* |
| **9a** ^b^ | 50 | 50 | 100 | 200 | 100 | >200 | 12.5 | 50 | 25 | >200 | 50 |
| **9b** | 200 | 200 | >200 | 200 | 200 | >200 | 50 | 100 | 100 | >200 | >200 |
| **9c** | >200 | >200 | 200 | 200 | >200 | >200 | 12.5 | >200 | >200 | >200 | >200 |
| **9d** | 6.3 | 100 | 100 | >200 | >200 | >200 | >200 | 50 | 6.3 | >200 | >200 |
| **9e** | >200 | 50 | 50 | >200 | >200 | 50 | >200 | 50 | 6.3 | >200 | >200 |
| **9f** | 3.13 | 12.5 | 25 | 50 | 25 | 6.3 | >200 | 12.5 | >200 | >200 | 12.5 |
| PA-824 | >200 | >200 | 200 | >200 | >200 | >200 | >200 | >200 | >200 | >200 | >200 |

*^a^Organisms abbreviated above are as follows: S.a., Staphylococcus aureus (ATCC 29213); MRSA, Staphylococcus aureus (NRS70); B.s., Bacillus subtilis (ATCC 23857); S.py, Streptococcus pyogenes (ATCC 700294); S.pn, Streptococcus pneumoniae (R6); E.f., Enterococcus faecalis (ATCC 33186); B.a., Bacillus anthracis sterne 34F2; B.c., Burkholderia cepacia (ATCC 25416); S.m., Stenotrophomonas maltophilia (ATCC 13637); E.c., Escherichia coli (ATCC 700926); E.c. ∆tolC,E. coli K12 ∆tolC. ^b^Compound 9a was inactive (MIC > 200 µg/mL) against Proteus mirabilis(ATCC 25933); Proteus vulgaris (ATCC 33420); Klebsiella pneumoniae (ATCC 33495); Acinetobacter baumannii (ATCC 19606); and Pseudomonas aeruginosa (PA01)1 are >200.*
