# Supplementary material for: Pentacyclic Nitrofurans with In Vivo Efficacy and Activity against Nonreplicating Mycobacterium tuberculosis
Source: PLoS One. 2014 Feb 5;9(2):e87909. doi: 10.1371/journal.pone.0087909 (PMC3914891; doi:10.1371/journal.pone.0087909)
Supplement: Table S2 — Assessment of Efficacy in an in vivo Murine Model of Acute Tuberculosis Infection. (DOCX) [file pone.0087909.s002.docx]

**Supporting Information – Table S2**

**Pentacyclic nitrofurans with *in vivo* efficacy and activity against latent *Mycobacterium tuberculosis***

Rakesh,^1†^ David F. Bruhn,^1†^ Michael S. Scherman,^2^ Lisa K. Woolhiser,^2^ Dora B. Madhura,^3^ Marcus M. Maddox,^1^ Aman P. Singh,^1,4^ Robin B. Lee,^1^ Julian G. Hurdle,^1‡^ Michael R. McNeil,^2^ Anne J. Lenaerts,^2^ Bernd Meibohm,^3^ Richard E. Lee^1,4*^

^1^ Department of Chemical Biology and Therapeutics, St. Jude Children’s Research Hospital, Memphis, TN, USA

^2^ Mycobacterial Research Laboratories, Department of Microbiology, Colorado State University, Fort Collins, CO, USA

^3^ Department of Pharmaceutical Sciences, College of Pharmacy, University of Tennessee Health Science Center, Memphis, TN, USA

^4^ Biomedical Sciences Program, Graduate Health Sciences, University of Tennessee Health Science Center, Memphis, TN, USA

^‡^ Present address: Department of Biology, University of Texas Arlington, Arlington, TX, USA
* [Richard.Lee@StJude.org](mailto:Richard.Lee@StJude.org) to whom all correspondence should be addressed.

† These authors contributed equally.

**Table S2: Assessment of Efficacy in an *in vivo* Murine Model of Acute Tuberculosis Infection**

1. Bacterial Load and Reduction in Lungs

| **Treatment Group** | **Log10CFU (SEM)** | **Log10CFU Reduction (SEM)** | **P Value** | **Statistically Significant?** |
| --- | --- | --- | --- | --- |
| Untreated | 8.07 (0.21) | - | - | - |
| 1 | 7.01 (0.11) | 1.06 (0.18) | < 0.05 | yes |
| 2 | 6.77 (0.14) | 1.30 (0.20) | < 0.05 | yes |
| 3 | 6.61 (0.11) | 1.47 (0.18) | < 0.05 | yes |
| 4 | 6.69 (0.07) | 1.38 (0.16) | < 0.05 | yes |
| 5 | 6.99 (0.06) | 1.08 (0.15) | < 0.05 | yes |
| 6 | 6.99 (0.15) | 1.10 (0.21) | < 0.05 | yes |
| 7 | 4.60 (0.21) | 3.47 (0.21) | < 0.05 | yes |

1. Bacterial Load and Reduction in Spleen

| **Treatment Group** | **Log10CFU (SEM)** | **Log10CFU Reduction (SEM)** | **P Value** | **Statistically Significant?** |
| --- | --- | --- | --- | --- |
| Untreated | 7.20 (0.14) | - | - | - |
| 1 | 6.25 (0.11) | 0.95 (0.18) | < 0.01 | yes |
| 2 | 5.72 (0.09) | 1.48 (0.17) | < 0.001 | yes |
| 3 | 5.43 (0.23) | 1.77 (0.27) | < 0.001 | yes |
| 4 | 6.03 (0.12) | 1.17 (0.18) | < 0.001 | yes |
| 5 | 5.76 (0.08) | 1.44 (0.16) | < 0.001 | yes |
| 6 | 6.17 (0.14) | 1.03 (0.20) | < 0.001 | yes |
| 7 | 2.61 (0.09) | 4.59 (0.16) | < 0.001 | yes |

CFU in organs indicated were enumerated plating serial dilutions of organ homogenate. Log_10_ protection was calculated by subtracting bacterial titer of each treatment from that of the untreated control. Mice were treated with 300 mg/kg QD with compounds **9a** dissolved in **(1)** 0.5% methylcellulose in DI-H_2_O **(2)** 30% Captisol in DI-H_2_O **(3)** 10% Vitamin E TPGS in DI-H_2_O **(4)** 0.5% Tween 80 in DI-H_2_O **(5)** 20% cyclodextrin in DI-H_2_O or **(6)** cold PEG (50:35:15 H_2_O:PEG300:PG) or (**7**) isoniazid dissolved in water. Error bars indicate SEM within treatment groups of 5-7 mice per group. Statistical significance was calculated by Tukeys’s multiple comparison test.
